# Independent Association of Circulating Vitamin D Metabolites with Anemia Risk in Patients Scheduled for Cardiac Surgery

**DOI:** 10.1371/journal.pone.0124751

**Published:** 2015-04-17

**Authors:** Jana B. Ernst, Tobias Becker, Joachim Kuhn, Jan F. Gummert, Armin Zittermann

**Affiliations:** 1 Clinic for Thoracic and Cardiovascular Surgery Heart and Diabetes Center North Rhine-Westphalia, Ruhr University Bochum, Bad Oeynhausen, Germany; 2 Institute for Laboratory and Transfusion Medicine, Heart and Diabetes Center North Rhine-Westphalia, Ruhr University Bochum, Bad Oeynhausen, Germany; University of Tennessee, UNITED STATES

## Abstract

**Background:**

Preoperative anemia is considered an independent risk factor of poor clinical outcome in cardiac surgical patients. Low vitamin D status may increase anemia risk.

**Methods:**

We investigated 3,615 consecutive patients scheduled for cardiac surgery to determine the association between preoperative anemia (hemoglobin [Hb] <12.5 g/dL) and circulating levels of the vitamin D metabolites 25-hydroxyvitamin D (25OHD) and 1,25-dihydroxyvitamin D (1,25[OH]_2_D).

**Results:**

Of the study cohort, 27.8 % met the criteria for anemia. In patients with deficient 25OHD levels (<30 nmol/l) mean Hb concentrations were 0.5 g/dL lower than in patients with adequate 25OHD levels (50.0–125 nmol/l; P<0.001). Regarding 1,25(OH)_2_D, mean Hb concentrations were 1.2 g/dL lower in the lowest 1,25(OH)_2_D category (<40 pmol/l) than in the highest 1,25(OH)_2_D category (>70 pmol/l; P<0.001). In multivariable–adjusted logistic regression analyses, the odds ratios for anemia of the lowest categories of 25OHD and 1,25(OH)_2_D were 1.48 (95%CI:1.19-1.83) and 2.35 (95%CI:1.86-2.97), compared with patients who had adequate 25OHD levels and 1,25(OH)_2_D values in the highest category, respectively. Anemia risk was greatest in patients with dual deficiency of 25OHD and 1,25(OH)_2_D (multivariable-adjusted OR = 3.60 (95%CI:2.40-5.40). Prevalence of deficient 25OHD levels was highest in anemia of nutrient deficiency, whereas low 1,25(OH)_2_D levels were most frequent in anemia of chronic kidney disease.

**Conclusion:**

This cross-sectional study demonstrates an independent inverse association between vitamin D status and anemia risk. If confirmed in clinical trials, preoperative administration of vitamin D or activated vitamin D (in case of chronic kidney disease) would be a promising strategy to prevent anemia in patients scheduled for cardiac surgery.

## Introduction

Anemia is a worldwide public health problem. With an estimated prevalence of 24.8%, it affects nearly one out of four of the global population [1]. The prevalence of anemia in patients undergoing cardiac surgery varies between 22.0% and 54.4% [2–5].

There is evidence from several observational studies that preoperative anemia is an independent predictor of poor clinical outcome in cardiac [2–4, 6–8] and non-cardiac [9] surgical patients. Different peri- and postoperative complications such as infections [3, 6, 7], stroke [2, 7] and renal failure [2, 7, 10], as well as prolonged ventilatory support [3, 7] and intensive care unit and in-hospital stay [3, 8, 9], were significantly associated with preoperative anemia. In addition, preoperative anemia is an independent risk factor of in-hospital and all-cause mortality [2, 6, 7, 9].

It has recently been shown that 25-hydroxyvitamin D (25OHD) deficiency is independently associated with lower hemoglobin (Hb) levels and higher anemia risk in patients scheduled for cardiac surgery [5]. The association between vitamin D deficiency and anemia is supported by various observational studies in different groups of patients [11–14]. Data in end-stage heart failure patients also indicate that circulating levels of the active vitamin D hormone, 1,25-dihydroxyvitamin D (1,25[OH]_2_D), is a better predictor of anemia than circulating 25OHD [14]. In line with these findings, first interventional studies in hemodialysis patients demonstrated an increase in Hb levels after intravenous administration of 1,25(OH)_2_D_3_, resulting in improved control of anemia [15, 16]. Experimental studies have provided evidence for potential mechanisms regarding how vitamin D may contribute to anemia prevention [17, 18]. Briefly, 1,25(OH)_2_D may influence bone marrow by stimulating erythropoiesis in red cell precursor cells via increased erythropoietin (EPO) sensitivity. Moreover, 1,25(OH)_2_D has the capacity to up-regulate proliferation of progenitor cells, independent of the presence of EPO.

The present study therefore aimed to investigate the association of preoperative 25OHD and 1,25(OH)_2_D levels with the risk of anemia in patients scheduled for cardiac surgery.

## Subjects and Methods

### Patients

For this cross-sectional study, we considered 3,852 adult patients who were scheduled for cardiac surgery at our institution between February 2012 and December 2013 (geographic latitude 52°N). We excluded 237 patients with incomplete data sets, e.g. missing 25OHD levels, 1,25(OH)_2_D levels and/or Hb levels, leaving a total of 3,615 patients for data analysis. The vast majority of patients were Caucasians. This investigation is a secondary analysis of the CALCITOP-study which was approved by the Ethics Committee Ruhr University Bochum at Bad Oeynhausen, was registered at clinicaltrials.gov as NCT02192528, and was recently published [19]. Participants provided their written confirmed consent. All patient information was anonymized and de-identified prior to analysis.

### Study Design

For analysis, we used routinely collected and filed demographic and clinical patient data (THGQIMS, Münster, Germany) and biochemical patient data (Lauris, SWISSLAB, Berlin, Germany). Blood samples were collected and analyzed on the last day before cardiac surgery. We assessed levels of 25OHD, 1,25(OH)_2_D and Hb, as well as other biochemical parameters (creatinine, C-reactive protein [CRP], leukocytes, erythrocytes, hematocrit [Htc], mean corpuscular volume [MCV], mean corpuscular hemoglobin [MCH], thrombocytes, red blood cell distribution width). In addition, we assessed patient characteristics like age, gender, season of blood sampling, body mass index, smoking, left ventricular ejection fraction [LVEF], concomitant diseases (myocardial infarction [MI], chronic obstructive pulmonary disease [COPD], stroke, hemofiltration, peripheral arterial occlusive disease [PAOD], hypertension, diabetes, hyperlipidemia) and medication use (diuretics, angiotensin-converting enzyme-inhibitors, angiotensin receptor-blockers, aspirin, clopidogrel, glucocorticoids).

### Biochemical Analysis

Circulating 1,25(OH)_2_D_3_ levels were measured by a liquid chromatography/tandem mass spectrometry method provided by Immundiagnostik (Bensheim, Germany). Since the detection limit of the method is 14 pmol/l, we considered values below this limit as 13.0 pmol/l. Circulating 25OHD levels (sum of 25[OH]D_2_ and 25[OH]D_3_) were analyzed by the autoanalyzer Liaison (DiaSorin, Stillwater, MN, USA). The measuring range was between 10 and 375 nmol/l. Values below the detection limit of 10.0 nmol/l were considered 9.9 nmol/l. CRP and creatinine levels were measured using the Architect Autoanalyzer (Abbott, Wiesbaden, Germany). Estimated glomerular filtration rate (eGFR) was calculated using the Modification of Diet in Renal Disease [20]. Blood Hb, Htc, erythrocytes, leukocytes, thrombocytes, MCV and MCH were measured by automated procedures using the Abbott CellDyn 3500 hematology analyzer (Abbott, Wiesbaden, Germany). According to earlier classifications [2, 5], Hb concentrations <12.5 g/dL were considered as anemic, which is the average threshold value of the World Health Organization’s gender-based definition (<13 g/dL in men and <12.0 g/dL in women). The following reference values were used for other hematological parameters: erythrocytes: males: 4.5–6.9 10^12^/l, females: 4.0–5.2 10^12^/l; Htc: males: 41–53%, females: 36–46%; MCH: 26–34 pg Hb/erythrocytes; MCV: 80–94 μm^3^. Together with low Hb levels, low MCV values can be an indicator of iron deficiency, whereas high MCV values can be an indicator of folate and/or vitamin B_12_ deficiency [21]. Therefore, we used earlier approaches to classify anemia hierarchically according to subtypes [5, 22]: anemia because of nutritional deficiency (iron deficiency: MCV <80 μm^3^; folate or vitamin B12 deficiency: MCV: >94 μm^3^), anemia of chronic kidney disease (CKD; eGFR: <60 ml/min per 1.73 m^2^), anemia of inflammation (CRP >10 mg/l) and unexplained anemia if none of these subtypes were present.

### Statistics

Categorical variables are reported as a percentage of observations. Continuous variables are presented as a mean and standard deviation. Clinical and demographic parameters of anemic and non-anemic patients were compared using Student´s *t* test, Chi-squared test and ANOVA. All continuous variables were normally distributed (Kolmogorov-Smirnov test). According to the Institute of Medicine [23], the following 25OHD cutoff values were used for classifying vitamin D status: deficiency (<30 nmol/l), inadequacy (30–49.9 nmol/l), adequacy (50–125 nmol/l) and potentially harmful (>125 nmol/l). Based on earlier classifications [14, 24], 1,25(OH)_2_D values were divided into three categories (<40 pmol/l; 40–70 pmol/l; >70 pmol/l).

To assess the independent association of 25OHD and 1,25(OH)_2_D categories with anemia, we performed logistic regression analysis (unadjusted and multivariable-adjusted). Results are expressed as odds ratios (ORs) with a 95% confidence interval. We considered the groups with adequate 25OHD levels (50–125 nmol/l) and 1,25(OH)_2_D levels >70 pmol/l as a reference group. The following covariates were included in multivariable models: model 1: unadjusted data; model 2: adjusted for age, gender and season of blood sampling (winter: January through March; spring: April through June; summer: July through September; fall: October through December); model 3: model 2 plus adjustments for body mass index, smoking, LVEF, concomitant diagnoses (MI, COPD, stroke, hemofiltration, PAOD, hypertension, diabetes and hyperlipidemia) and medications (diuretics, angiotensin-converting enzyme-inhibitors, angiotensin receptor-blockers, aspirin, clopidogrel, glucocorticoids); model 4: model 3 plus adjustments for kidney function (eGFR in ml/min per 1.73 m^2^) and inflammatory processes (CRP in mg/dL). In sensitivity analysis, we also used the following 25OHD classification: <50 nmol/l, 50–125 nmol/l and >125 nmol/l. We considered the P-values <0.05 as statistically significant. P-values are two-sided. Analyses were performed using SPSS version 21.0 (SPSS, Inc., Chicago, IL, USA).

## Results

Of the study cohort, 26.1% were 25OHD deficient (<30.0 nmol/l), 35.4% had inadequate 25OHD values (30–49.9 nmol/l), 36.7% had adequate 25OHD values (50–125 nmol/l) and 1.7% had values >125 nmol/l. Regarding 1,25(OH)_2_D, 29.4% had values below 40 pmol/l, 40.6% values between 40 and 70 pmol/l and 30.0% had values >70 pmol/l. Baseline characteristics of the study cohort are listed in Table 1, broken down by anemia status. Of the study cohort, 27.8% met the criteria for anemia. Compared with non-anemic patients, circulating 25OHD and 1,25(OH)_2_D levels were significantly lower in anemic patients. Anemic patients were significantly older, more frequently female but less often smokers than non-anemic patients. In addition, anemic patients had significantly lower LVEF values and suffered more often from COPD, hemofiltration, PAOD, diabetes and hyperlipidemia than non-anemic patients. In addition, anemia was associated with reduced Hb, Htc, MCV, MCH and erythrocyte values, but MCV, MCH and erythrocyte values were still within the respective reference range. CRP was significantly elevated in the anemic group.

**Table 1:** Characteristics of the study cohort by anemia status.

	Anemic	Non-anemic	*P* value
	(<12.5 g/dl)	(≥12.5 g/dl)	
	n = 1,006	n = 2,609	
Age (years)	72±10.7	67±11.1	<0.001
Gender (% males)	47.5	74.7	<0.001
BMI (kg/m^2^)	27.5±5.3	27.8±4.3	0.114
Smokers (%)	21.2	31.8	<0.001
LVEF (%)	56.3±12.5	57.8±12.1	0.001
Concomitant diseases			
MI (%)	19.2	16.8	0.096
COPD (%)	11.2	7.8	0.001
Stroke (%)	6.3	4.6	0.051
Hemofiltration (%)	5.3	0.4	<0.001
PAOD (%)	9.7	6.6	0.002
Hypertension (%)	80.9	78.8	0.183
Diabetes (%)	34.6	22.9	<0.001
Hyperlipidemia (%)	62.6	66.4	0.035
Medications			
Diuretics (%)	57.8	36.7	<0.001
ACE-Inhibitors (%)	47.6	45.2	0.192
AT-blockers (%)	12.9	11.4	0.206
Aspirin (%)	50.7	44.9	0.002
Clopidogrel (%)	9.2	6.7	0.013
Glucocorticoids (%)	4.0	2.9	0.114
Biochemical parameters			
eGFR (ml/min/1.73 m^2^)	64.1±26.2	78.6±20.6	<0.001
CRP (mg/dl)	2.3±4.7	0.7±1.6	<0.001
25OHD (nmol/l)	46.9±30.4	49.3±26.0	0.026
1,25(OH)_2_D (pmol/l)	46.7±26.8	60.6±27.5	<0.001
Hemoglobin (g/dl)	11.1±1.1	14.3±1.1	<0.001
Leukocytes (10^9^/l)	8.2±3.8	7.8±3.1	0.003
Erythrocytes (10^12^/l)	3.8±0.5	4.6±0.4	<0.001
Hematocrit (%)	33.5±3.2	41.9±3.3	<0.001
MCV (μm^3^)	89.3±6.3	90.4±4.5	<0.001
MCH (pg Hb/red blood cell)	29.6±2.6	30.8±1.8	<0.001
Thrombocytes (10^9^/l)	249±100.8	231±72.7	<0.001
RDW (%)	14.0±2.2	12.4±1.2	<0.001

ACE = Angiotensin-converting-enzyme; AT = Angiotensin receptor; BMI = Body Mass Index; COPD = chronic obstructive pulmonary disease; CRP = C-reactive protein; eGFR = estimated globular filtration rate; Hb = Hemoglobin; LVEF = Left ventricular ejection fraction; MCH = mean corpuscular/cellular hemoglobin; MCV = mean corpuscular/cell volume; MI = myocardial infarction; PAOD = peripheral artery occlusive disease; RDW = Red Blood Cell Distribution Width ([S_v_ x 100]/MCV)

Tables 2 and 3 illustrate the hematological parameters broken down by 25OHD status and 1,25(OH)_2_D categories, respectively. Anemia risk was greatest in the groups with the lowest 25OHD and 1,25(OH)_2_D concentrations. In detail, mean Hb concentrations were 0.5 g/dL lower in patients with deficient levels of 25OHD levels (<30 nmol/l) than in patients with adequate 25OHD levels (50.0–125 nmol/l; P<0.001). Regarding 1,25(OH)_2_D, mean Hb concentrations were 1.2 g/dL lower in patients in the lowest 1,25(OH)_2_D category (<40 pmol/l) than in the highest 1,25(OH)_2_D category (>70 pmol/l; P<0.001). Hematological parameters, such as erythrocytes, Htc, MCH and MCV, were all lowest in patients with low 1,25(OH)_2_D levels. MCV and MCH were lowest in patients with deficient 25OHD levels, whereas erythrocytes and Htc were lowest in patients with 25OHD levels >125 nmol/l. MCV differed significantly between 25OHD categories, but not between 1,25(OH)_2_D categories.

**Table 2:** Hematological parameters by cutoffs of 25-Hydroxyvitamin D.

25OHD	<30 nmol/l	30–49.9 nmol/l	50–125 nmol/l	>125 nmol/l	*P* value
	n = 945	n = 1281	n = 1328	n = 61	for trend
Hemoglobin (g/dl)	13.0±2.0	13.5±1.8	13.5±1.7	12.8±1.8	<0.001
Erythrocytes (10^12^/l)	4.3±0.6	4.4±0.6	4.4±0.6	4.2±0.5	<0.001
Hematocrit (%)	38.7±5.4	39.8±5.0	39.9±4.6	38.2±4.8	<0.001
MCV (μm^3^)	89.7±5.9	90.2±4.9	90.3±4.5	90.8±5.9	0.034
MCH (pg Hb/Erythrocytes)	30.2±2.5	30.6±2.0	30.6±1.8	30.4±2.5	<0.001

Hb = Hemoglobin; MCH = mean corpuscular/cellular hemoglobin; MCV = mean corpuscular/cell volume

**Table 3:** Hematological parameters by cutoffs of 1,25-Dihydroxyvitamin D.

1,25(OH)_2_D	<40 pmol/l	40–70 pmol/l	>70 pmol/l	*P* value
	n = 1064	n = 1468	n = 1083	for trend
Hemoglobin (g/dl)	12.7±1.9	13.5±1.7	13.9±1.6	<0.001
Erythrocytes (10^12^/l)	4.2±0.6	4.4±0.6	4.6±0.5	<0.001
Hematocrit (%)	37.6±5.3	39.9±4.8	40.8±4.3	<0.001
MCV (μm^3^)	89.9±5.6	90.3±4.9	90.0±4.8	0.101
MCH (pg Hb/Erythrocytes)	30.2±2.3	30.6±2.0	30.6±2.0	<0.001

Hb = Hemoglobin; MCH = mean corpuscular/cellular hemoglobin; MCV = mean corpuscular/cell volume

In Tables 4 and 5, the ORs for anemia are given by categories of 25OHD and 1,25(OH)_2_D. In the unadjusted model, the ORs for patients in the lowest categories of 25OHD and 1,25(OH)_2_D were significantly higher = 1.79 (95% CI: 1.49–2.15) and 3.73 (95% CI: 3.05–4.56), compared with the respective reference group. In the fully adjusted models, the ORs for anemia were attenuated, but remained significant. Compared with the respective reference group, the OR for the category of 25OHD deficient patients was = 1.48 (95% CI: 1.19–1.83) and was = 2.35 (95% CI: 1.86–2.97) for the lowest 1,25(OH)_2_D category.

**Table 4:** Unadjusted and adjusted odds ratio (OR) for anemia by cutoffs of 25-Hydroxyvitamin D.

25OHD	N	Anemia (%)	Model 1	Model 2	Model 3	Model 4
nmol/l			OR (95% CI)	OR (95% CI)	OR (95% CI)	OR (95% CI)
<30	945	337 (35.7)	1.79 (1.49–2.15)	1.63 (1.34–1.98)	1.50 (1.22–1.84)	1.48 (1.19–1.83)
30–49.9	1281	331 (25.8)	1.13 (0.94–1.34)	1.06 (0.88–1.28)	1.06 (0.87–1.28)	1.05 (0.85–1.28)
50–125	1328	314 (23.6)	1.0 (reference)	1.0 (reference)	1.0 (reference)	1.0 (reference)
>125	61	24 (39.3)	2.10 (1.23–3.56)	2.27 (1.30–3.96)	1.69 (0.92–3.10)	1.34 (0.71–2.52)

Model 1: unadjusted

Model 2: adjusted for age, gender and season of blood drawing

Model 3: adjusted as in model 2 and for concomitant diseases, medications, smoking, body mass index and left ventricular ejection fraction

Model 4: adjusted as in model 3 and for kidney function (eGFR) and inflammatory process (CRP)

**Table 5:** Unadjusted and adjusted odds ratio (OR) for anemia by cutoffs of 1,25-Dihydroxyvitamin D.

1,25(OH)_2_D	N	Anemia (%)	Model 1	Model 2	Model 3	Model 4
pmol/l			OR (95% CI)	OR (95% CI)	OR (95% CI)	OR (95% CI)
<40	1064	452 (42.5)	3.73 (3.05–4.56)	3.50 (2.83–4.33)	2.97 (2.38–3.71)	2.35 (1.86–2.97)
40–70	1468	375 (25.5)	1.73 (1.42–2.11)	1.69 (1.37–2.08)	1.60 (1.29–1.98)	1.52 (1.22–1.89)
>70	1083	179 (16.5)	1.0 (reference)	1.0 (reference)	1.0 (reference)	1.0 (reference)

Model 1: unadjusted

Model 2: adjusted for age, gender and season of blood drawing

Model 3: adjusted as in model 2 and for concomitant diseases, medications, smoking, body mass index and left ventricular ejection fraction

Model 4: adjusted as in model 3 and for kidney function (eGFR) and inflammation (CRP)

In patients with dual deficiency of 25OHD and 1,25(OH)_2_D (n = 336), the fully adjusted OR for anemia was compared with patients who had adequate 25OHD levels and 1,25(OH)_2_D levels in the highest category (n = 438) = 3.60 (95% CI: 2.40–5.40; P<0.001). In sensitivity analysis, the fully adjusted OR of patients with 25OHD values <50 nmol/l was = 1.21 (95% CI: 1.01–1.45), compared with the reference group of 50–125 nmol/l.

**Fig 1:**
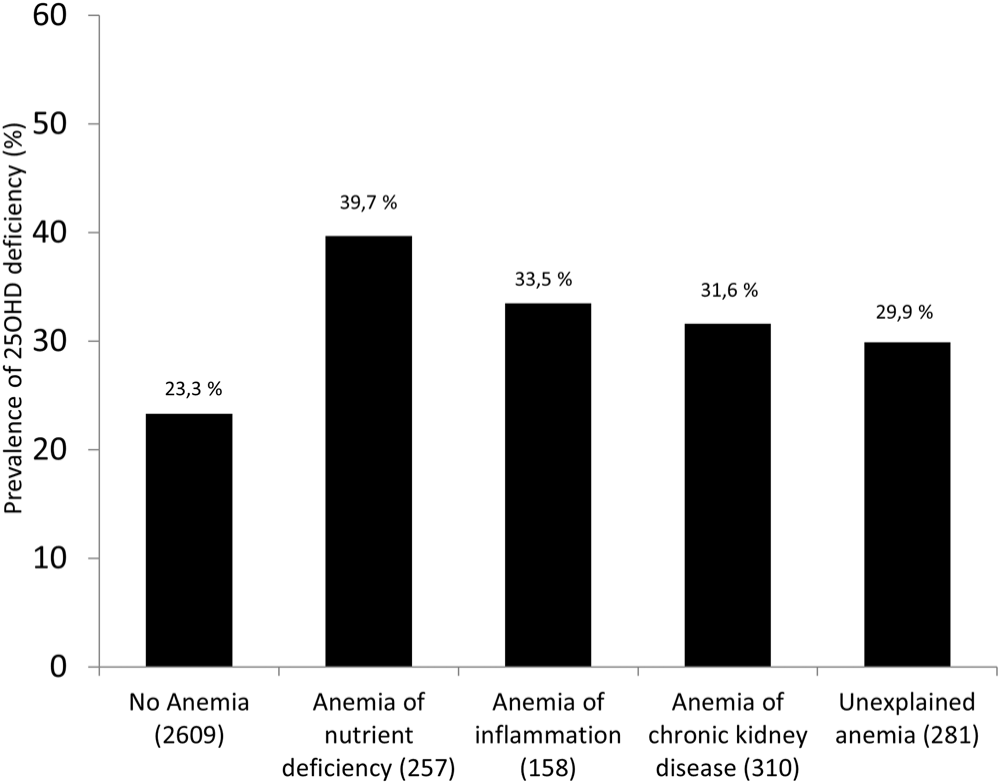
Prevalence of deficient 25OHD levels according to subtypes of anemia. There were significant differences between subgroups (*P*<0.001). Number of patients in brackets.

**Fig 2:**
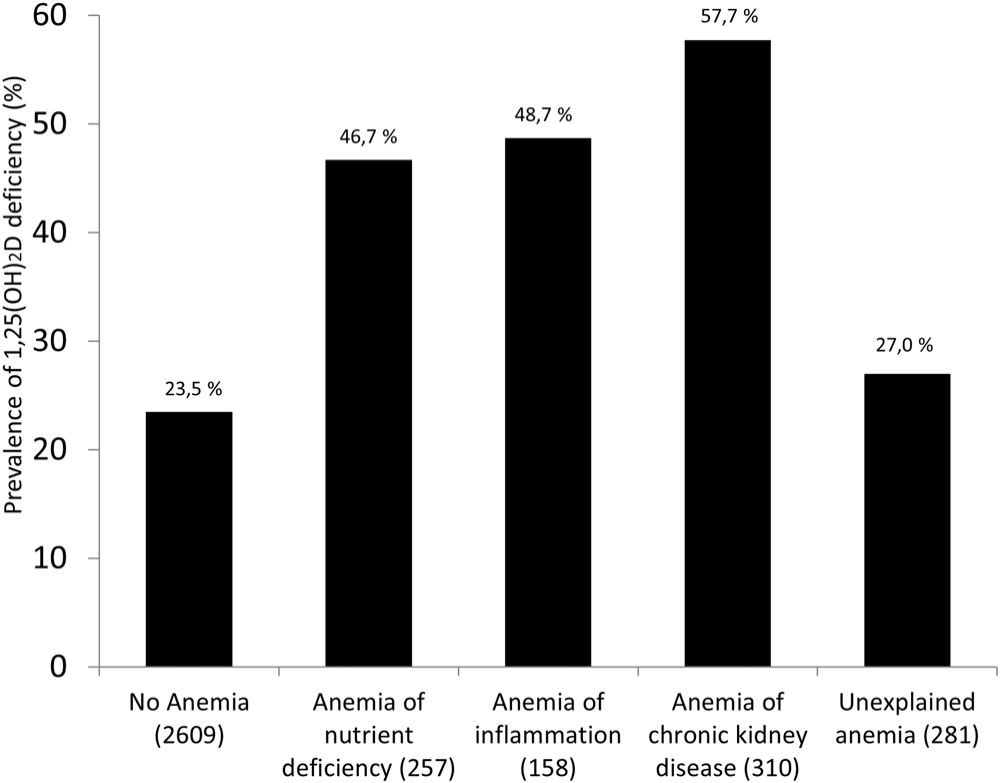
Prevalence of 1,25(OH)2D levels <40 pmol/l according to subtypes of anemia. There were significant differences between subgroups (*P*<0.001). Number of patients in brackets.

The prevalence of deficient 25OHD levels was highest in anemia of nutrient deficiency, whereas low 1,25(OH)_2_D levels were most frequent in anemia of CKD (Figs 1 and 2).

## Discussion

This cross-sectional study demonstrates that low 25OHD and 1,25(OH)_2_D levels are independently associated with low Hb levels and anemia risk in patients scheduled for cardiac surgery. Patients with 25OHD values <30 nmol/l and 1,25(OH)_2_D values <40 pmol/l had the highest risk for anemia, whereas the risk was lowest in patients with adequate 25OHD levels (50–125 nmol/l) and 1,25(OH)_2_D levels above 70 pmol/l. Circulating 1,25(OH)_2_D was a better predictor of anemia than circulating 25OHD.

Our data confirm earlier studies of an independent inverse association between circulating 25OHD and anemia risk [5, 11, 12, 22, 25–30]. However, the association of 1,25(OH)_2_D with anemia risk has rarely been studied, and the few available studies on this topic have primarily been performed in patients with CKD and end-stage heart failure [13] [14]. In end-stage heart failure [14], Hb concentrations were 1.6 g/dL higher in patients with values >70 pmol/l compared to patients with values <40 pmol/l. These earlier findings are in general agreement with the data of the present study.

There are several suggested causes why low 1,25(OH)_2_D may play a role in the development of anemia. Vitamin D modulates the level of systemic cytokine production. Therefore, vitamin D may reduce the inflammatory milieu that leads to anemia of chronic disease [11]. In addition, vitamin D may influence folate and iron absorption by increasing intestinal proton-coupled folate transporter [31] [32]. Iron participates in the renal activation of 25OHD into 1,25(OH)_2_D [33]; the enzymatic conversion is catalyzed by a cytochrome P450-dependent ferrodoxin reductase [34]. 1,25(OH)_2_D can also stimulate erythropoiesis in red blood cell precursor cells by increasing EPO sensitivity and can up-regulate proliferation of progenitor cells [17, 18]. First interventional studies support the hypothesis that 1,25(OH)_2_D administration and probably also high-dose bolus application of vitamin D is effective in enhancing Hb levels. Briefly, two studies in patients with CKD demonstrated an increase in mean Hb concentration after 12 months of intravenous 1,25(OH)_2_D administration by 1.0 mg/dL [15] and 1.2 mg/dL [16], and therefore improved control of anemia with reduced need for EPO. However, results must be considered preliminary since no control group was included in the two studies. In another study in hemodialysis patients, intravenous administration of 1,25(OH)_2_D decreased the weekly EPO dose requirement by 50% [35]. Similarly, high-dose monthly oral vitamin D_2_ administration (50,000 IU) was associated with non-significant [36] and significant [37] reductions in doses of erythropoiesis-stimulating agents (ESA), while mean Hb levels remained unchanged. Interestingly enough, it has been demonstrated that high-dose oral bolus administration of vitamin D is even able to increase circulating 1,25(OH)_2_D levels in CKD patients [38].

Data obtained in the United States demonstrate that blood transfusion is the most common procedure performed during hospitalization (11.0% of hospital stays with a procedure) [39]. With a range of 35.8% to 60.0%, the transfusion rate in patients undergoing a cardiovascular procedure is very high [40–46]. However, the use of blood transfusion involves several risks. Blood transfusion is an independent risk factor for clinical complications like prolonged ventilatory support [3, 7], increased length of hospital stay [45, 46], infection [43–45, 47] and mortality [40–43, 45]. The increased risk of blood transfusion has been attributed to the presence of leucocytes in allogeneic blood transfusion thereby causing immunomodulatory events [48]. In addition, duration of blood storage influences clinical outcome with higher incidence of prolonged mechanical ventilation, renal failure, sepsis and in-hospital mortality in patients receiving older blood products (>14 days) [49]. Besides the use of leukocyte-depleted red blood cells and short storage time, reduction or even avoidance of the use of blood transfusion might be another interesting approach. Anemia is the most important risk factor for transfusion [50]. If confirmed in randomized controlled trials, administration of vitamin D or its active form 1,25(OH)_2_D could be a promising preventive or therapeutic option to decrease the prevalence of anemia in elective cardiac surgical patients. Of note, CKD patients (stages IV and V) should preferably be treated with activated vitamin D.

In our study, prevalence of deficient 25OHD levels was highest in anemia of nutrient deficiency, whereas low 1,25(OH)_2_D levels were most frequent in anemia of chronic kidney disease. With regard to CKD, this may be due to low GFR, resulting in reduced 1,25(OH)_2_D synthesis. However, it may also be that CKD-induced increased 24-hydroxylase activity [51] leads to enhanced conversion of 25OHD and 1,25(OH)_2_D into their 24-hydroxylated inactive forms. It is well known that 1,25(OH)_2_D levels are suppressed in CKD. However, low 1,25(OH)_2_D levels were also present in anemia of inflammation and anemia of nutrient deficiency. It has recently been shown that low 1,25(OH)_2_D levels are independently associated not only with poor kidney function but also with low 25OHD levels, high CRP levels, diabetes mellitus and high values of the cardio-surgical risk marker EuroSCORE [19]. It may therefore well be that the anemia subtype ‘nutrient deficiency’ may only be a surrogate for poor health status and that circulating 1,25(OH)_2_D levels are also suppressed by other, currently unknown factors.

Our study has some strengths but also some limitations. Its strengths are measurement of the active vitamin D hormone, 1,25(OH)_2_D, in a large cohort of patients and adjustments for several demographical and clinical variables. One inherent limitation is the observational study design, which prevents us from concluding that the association between 25OHD and 1,25(OH)_2_D deficiency and anemia is causal. It is another limitation that we were unable to assess the use of ESA or to measure EPO concentrations. However, since the association of low 1,25(OH)_2_D levels with anemia was highest in anemia of CKD, it is rather unlikely that ESA were frequently used in our study cohort. Finally, no data on parathyroid hormone, magnesium or fibroblast growth factor 23 levels were available, parameters that are involved in 1,25(OH)_2_D regulation and therefore play a central role in vitamin D metabolism.

In conclusion, our study demonstrates that 25OHD and 1,25(OH)_2_D levels are independently associated with low Hb concentrations and high anemia risk in cardiac surgical patients. Circulating 1,25(OH)_2_D was a better predictor of anemia than circulating 25OHD. Future studies have to clarify whether the association is causal and whether preoperative administration of vitamin D or 1,25(OH)_2_D is able to successfully prevent or treat anemia in patients scheduled for cardiac surgery.
